# Clinical and epidemiological characterization of demodicosis cases in the pediatric population at the Hospital Clinic of the University of Chile (2013‒2020)^☆^

**DOI:** 10.1016/j.abd.2023.10.008

**Published:** 2024-11-17

**Authors:** Claudia Schroder, Matías Gárate, Diego Orlandi, Ligia Aranibar, Francisco Silva

**Affiliations:** a Department of Dermatology, Hospital Clínico Universidad de Chile, Santiago, Chile; b Dermatology Service, Hospital Clínico Universidad de Chile, Santiago, Chile; c Clinical Laboratory Service, Microbiology Unit, Hospital Clínico Universidad de Chile, Santiago, Chile

Dear Editor,

*Demodex folliculorum* and *Demodex brevis* are commensal mites of the pilosebaceous unit, and their presence in children under 18 years old is rarely described, this can be explained by the low production of sebum in childhood.^1^, ^2^ It is believed that their pathogenic role occurs when they penetrate the dermis, multiplying and causing local inflammation.^3^ The term demodicosis refers to the proliferation of this mite when it is associated with inflammatory skin pathology, mainly in the form of papulopustular rosacea or blepharitis, although it can also present as pruritic eczematous plaques and perioral dermatitis.^4^

It is important to detect this uncommon pathology in the pediatric population due to its association with immunosuppression, such as malnutrition, HIV, or the presence of hematological malignancy. The diagnosis is clinical, and it is confirmed by direct microscopy of scraping of a skin lesion.^5^

Regarding national and international literature, there is little description of the presence of these mites in children under 18 years of age. For this reason, there is no consensus on its treatment and follow-up, as well as the inflammatory or chronic implications that this pathology could imply.

Our objective is to clinically characterize patients under 18 years old with a diagnosis of demodicosis.

A retrospective descriptive study was made, based on clinical records of pediatric patients with positive tests for *Demodex* performed by a specialized laboratory technician between 2013 and 2020.

From 2013 to 2020 we analyzed 2,491 mite tests in the facial area positive for *Demodex*, of which 94 were from patients under 18 years of age. Finally, only 45 patients with complete clinical information were included. From these patients, information on age, gender, morbid history, and number of mites per lesion analyzed was collected. Additionally, the presence of erythema, papules, pustules, and peeling on the skin was analyzed. The presence of ocular and other involvement (rhinophyma, erosion, nodules, stye and conjunctivitis) was investigated.

Regarding the treatment of the patients, this was classified as topical and/or systemic treatment, depending on what they received.

The average age of the patients studied was 9.3 years, with a standard deviation of 4.1 years. 17.8% (8 patients) were male and 82.2% (37 patients) were female (Table 1). Regarding the number of *Demodex* per mite test performed, the average was 28.4 *Demodex* per sample, with a minimum of 2 and a maximum of 98 *Demodex*. Regarding the symptoms and clinical signs present in the patients, we observed (Table 2): papules (97.8%), erythema (95.6%), pustules (55.6%) and peeling (28.9%). The presence of other symptoms also stands out, such as stye (11.1%), erosion (8.9%), rhinophyma (6.6%) and conjunctivitis (4.4%). Among the patients' morbid history, the following stand out acne (4 patients), asthma (3 patients), rhinitis (2 patients), psoriasis (1 patient) and Netherton syndrome (1 patient). In Figs. 1 and 2, the clinical symptoms of the patients can be seen, with papules and erythema being the most frequent findings.

**Table 1:** Demographic characteristics and number of mites per sample.

	Average	Standard deviation
Age	9.3 years	± 4.1 years
Number of mites per sample	28.4 mites	‒
Gender	%	n
Male	17.8	8
Female	82.2	37
Total	100	45

**Table 2:** Clinical characteristics of pediatric patients expressed in percentage and total number.

	%	n
Phototype^a^		
I	0	0
II	23.7	9
III	63.2	24
IV	13.2	5
V	0	0
VI	0	0
Papules	97.8	44
Erythema	95.6	43
Pustules	55.6	25
Flaking	28.9	13
Others		
Rhinophyma	2	4.4
Nodule and rhinophyma	1	2.2
Erosión	4	8.9
Ocular		
Stye	11.1	5
Conjunctivitis	4.4	2
Morbid history		
Acne	8.9	4
Asthma	6.7	3
Allergic rinitis	4.4	2
Psoriasis	2.2	1
Netherton syndrome	2.2	1

a As for the phototype, it was only described in 38 patients. In the rest, the % is with respect to a total “n” of 45 patients.

**Fig. 1:**
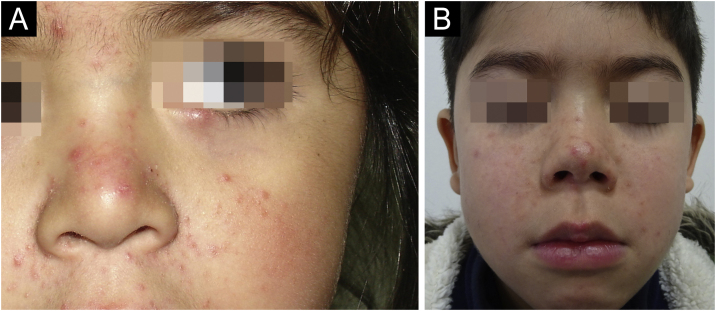
(A) 4-year-old girl with erythema, papules and pustules in the facial area; (B) 8-year-old boy with basal erythema and papules in the facial area.

**Fig. 2:**
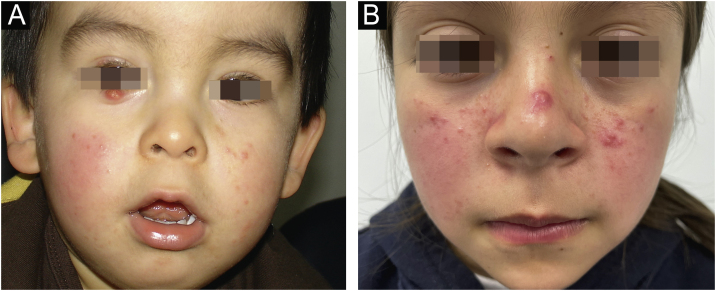
(A) 1-year-old child with erythema and micropapules on cheeks. An erythematous- yellowish nodule on the free edge of the right lower eyelid stands out as the main manifestation of demodicosis. (B) 9-year-old girl with centrofacial erythema associated with a significant number of papules and pustules in the facial area.

In the matter of the diagnosis recorded in the files, this is varied and includes demodicosis, rosacea, corticosteroid dependence and perioral dermatitis.

The treatment used was only described in 41 patients: 83% received exclusively topical treatment and 17% associated with systemic treatment.

Initially, pediatric demodicosis was mainly described in patients with immunosuppression, but currently, cases have been described in children without a morbid history.^5^, ^6^, ^7^, ^8^, ^9^, ^10^ In the present case series there is only one child with immunosuppression despite finding some patients with the use of inhaled corticosteroids, such as those with obstructive respiratory pathology (3 patients with asthma and 2 patients with allergic rhinitis). This could thus be a factor that influences a greater predisposition to demodicosis.

The cause of clinical findings in *Demodex* infestation is still unknown. Although demodicosis has been demonstrated in several skin conditions, the role of *Demodex* mites in dermatologic conditions is still controversial.^11^

In the present study of Chilean pediatric patients, the symptoms and signs, in addition to the clinical history, are similar and comparable to international studies.^4^ This is how the main symptoms and signs point to secondary demodicosis, with an increase in papulopustular rosacea or ocular and perioral dermatitis. As to the comorbidities, only 42% had any, which ranged from inflammatory diseases such as psoriasis and hidradenitis suppurativa, to genetically based pathologies such as Netherton syndrome.

When carrying out the study, the small number of mite test examinations requested for children under 18 years of age is striking. The latter would respond to the low suspicion of demodicosis in children and avoid uncomfortable procedures for parents and children, thus losing the opportunity to expand the differential diagnosis. Finally, with regard to treatment, in our series of cases, very varied regimens were observed, which included the agents classically used in this pathology to associated regimens such as zinc oxide or alpha-bisabolol. In respect of oral therapy, the main agent used is oral doxycycline or lymecycline for periods ranging from 4 to 8 weeks. It should be considered that within the treatment we have two therapeutic agents with age restrictions for their administration. For this reason, our therapeutic arsenal may be limited to severe demodicosis in very young patients.

Our series is the first reported in the Chilean pediatric population and allows us to propose guidelines for future studies, evaluate treatments, recurrences, and disseminate in the medical community the possible pathogenic role of *Demodex spp*. in patients of this age. This disease is rare in the pediatric population, so we propose that demodicosis should be suspected in all children with papulopustular eruptions on the face or in cases of refractory facial inflammatory disorders, such as periorificial dermatitis and infantile rosacea, in which case a mite test or other diagnostic test should be made in order to confirm the diagnosis for appropriate treatment.

As regards the limitations of our study, it corresponds to a descriptive study, with a low number of patients and without long-term post-treatment follow-up.

## Authors’ contributions

Claudia Schroder: Writing of the manuscript or critical review of important intellectual content; critical review of the literature; final approval of the final version of the manuscript.

Matías Gárate: The study concept and design; data collection, or analysis and interpretation of data; statistical analysis; writing of the manuscript or critical review of important intellectual content; data collection, analysis and interpretation.

Diego Orlandi: The study concept and design; data collection, or analysis and interpretation of data; statistical analysis; writing of the manuscript or critical review of important intellectual content; data collection, analysis and interpretation.

Ligia Aranibar: Effective participation in the research guidance; intellectual participation in the propaedeutic and/or therapeutic conduct of the studied cases.

Francisco Silva: Effective participation in the research guidance; intellectual participation in the propaedeutic and/or therapeutic conduct of the studied cases.

## Financial support

None declared.

## Conflicts of interest

None declared.
